# *In Silico* Docking of Forchlorfenuron (FCF) to Septins Suggests that FCF Interferes with GTP Binding

**DOI:** 10.1371/journal.pone.0096390

**Published:** 2014-05-02

**Authors:** Dimitrios Angelis, Eva Pauline Karasmanis, Xiaobo Bai, Elias T. Spiliotis

**Affiliations:** Department of Biology, Drexel University, Philadelphia, Pennsylvania, United States of America; NHLBI, NIH, United States of America

## Abstract

Septins are GTP-binding proteins that form cytoskeleton-like filaments, which are essential for many functions in eukaryotic organisms. Small molecule compounds that disrupt septin filament assembly are valuable tools for dissecting septin functions with high temporal control. To date, forchlorfenuron (FCF) is the only compound known to affect septin assembly and functions. FCF dampens the dynamics of septin assembly inducing the formation of enlarged stable polymers, but the underlying mechanism of action is unknown. To investigate how FCF binds and affects septins, we performed *in silico* simulations of FCF docking to all available crystal structures of septins. Docking of FCF with SEPT2 and SEPT3 indicated that FCF interacts preferentially with the nucleotide-binding pockets of septins. Strikingly, FCF is predicted to form hydrogen bonds with residues involved in GDP-binding, mimicking nucleotide binding. FCF docking with the structure of SEPT2-GppNHp, a nonhydrolyzable GTP analog, and SEPT7 showed that FCF may assume two alternative non-overlapping conformations deeply into and on the outer side of the nucleotide-binding pocket. Surprisingly, FCF was predicted to interact with the P-loop Walker A motif GxxxxGKS/T, which binds the phosphates of GTP, and the GTP specificity motif AKAD, which interacts with the guanine base of GTP, and highly conserved amino acids including a threonine, which is critical for GTP hydrolysis. Thus, *in silico* FCF exhibits a conserved mechanism of binding, interacting with septin signature motifs and residues involved in GTP binding and hydrolysis. Taken together, our results suggest that FCF stabilizes septins by locking them into a conformation that mimics a nucleotide-bound state, preventing further GTP binding and hydrolysis. Overall, this study provides the first insight into how FCF may bind and stabilize septins, and offers a blueprint for the rational design of FCF derivatives that could target septins with higher affinity and specificity.

## Introduction

Septins are a family of guanosine triphosphate (GTP) binding proteins, which are highly conserved in fungal and metazoan organisms [1], [2]. Septins belong to the superclass of P-loop GTPases, which structurally are characterized by alternating alpha helices and beta strands separated by flexible loops [3], [4]. All septins contain the GTP-specificity motif AKAD, which interacts with the guanine base of GTP, and the Walker A (GxxxxGKS/T) and B (DxxG) motifs that bind to the phosphate groups of GTP and coordinate with a magnesium ion for GTP hydrolysis, respectively [4], [5]. In contrast to the monomeric small GTPases of the Ras superfamily, septins oligomerize via their GTP-binding domains, forming filamentous hetero-polymers [6], [7].

Next to microtubules, actin microfilaments and intermediate filaments, septins represent a fourth cytoskeleton-like element, which plays essential roles in many cellular functions by controlling the localization of cellular proteins [8]–[10]. Similar to microtubules and actin, septin filament assembly and disassembly involves nucleotide binding and hydrolysis, but how this occurs is not well understood. Septins polymerize by interacting with one another via the N- and C-termini (NC interface) and the nucleotide-binding pocket (G interface) of their GTP binding domains [6]. Structural studies indicate that GTP-binding triggers conformational changes that destabilize the NC interface, while GTP hydrolysis appears to have the opposite effect on the G interface, which is more stable in its GDP- than GTP-bound state [11]–[14]. Septin monomers hydrolyze GTP faster than septin dimers and oligomers, whose nucleotide-binding pocket is not readily accessible by GTP [12], [15]. Not all septins, however, are capable of hydrolyzing GTP. Septins that lack a threonine residue, which is critical for GTP hydrolysis, are constitutively bound to GTP and septin hetero-hexamers contain both GDP and GTP at a ratio of 2∶1 [6], [11]. Interestingly, *in vitro* biochemical studies of septin assembly have shown that GTP-gamma-S, a slowly hydrolysable GTP analog, promotes the assembly of septin monomers into homo-polymeric filaments [16]. While more work is needed to fully understand how GTP-binding and hydrolysis affects the dynamics of septin filament assembly, pharmacological agents that stabilize or depolymerize septin filaments can be useful tools in understanding the mechanisms of septin assembly and function.

To date, forchlorfenuron (FCF; *N*-(2-Chloro-4-pyridyl)-*N′*-phenylurea; CPPU) is the only small molecule compound known to affect septin filament assembly. FCF is a synthetic plant cytokinin that consists of a chlorinated pyridine and a phenol ring joined together by a urea group [17]. FCF was fortuitously discovered to reversibly affect the localization and morphology of septins in the budding yeast *Saccharomyces cerevisiae* [18]. Subsequent studies in mammalian cells and the filamentous fungus *Ashbya gossypii* showed that FCF dampens the dynamics of septin filaments, amplifying the length and thickness of septin filaments [19], [20]. Notably, FCF had a similar effect in a cell-free *in vitro* assay, boosting the assembly of purified recombinant septin complexes into higher order filamentous structures [19]. While not ruling out the possibility of off-target effects in cells, these studies suggest that FCF has a direct effect on septin assembly. Understanding how FCF binds and affects septins can provide new insights into the mechanism of septin polymerization and guide the design of small molecule compounds that target septins with high specificity and affinity.

Computational simulations of drug-target interactions using *in silico* molecular docking and molecular dynamics approaches are commonly used for the rational design and screening of drugs [21], [22]. As evidenced by the paucity of high-resolution atomic-level septin structures, studying septin-FCF interactions by X-ray crystallography can be very challenging. To gain an insight into how FCF binds septins, we performed *in silico* simulations of FCF docking to all available high-resolution crystal structures of septins. The results indicate that FCF interacts preferentially with the nucleotide-binding pocket of septins, forming bonds with amino acids and atoms involved in GTP-binding and hydrolysis. Collectively, our studies suggest that FCF mimics nucleotide-binding and thereby, interferes with the GTP-binding dynamics and turnover that underlie the assembly of filamentous septins.

## Materials and Methods

### Plasmids

His-SEPT2-expressing plasmid was constructed by PCR amplifying mouse SEPT2 (NP_006146) using the primers 5′- TCGGGATCCATGTCTAAGCAACAACC-3′ and 5′- ATCCTCGAGTCACACATGCTGCCCG-3′ and cloning the amplified fragment into the BamHI and XhoI sites of pET-28a(+). His-SEPT2 sequence and its upstream ribosome binding site was then excised using XbaI and XhoI, and subcloned into pET-15b, which contains an ampicillin resistance marker. His-SEPT3 was constructed by amplifying the SEPT3 (NM_145733) sequence from a pcDNA3.0 Flag-tagged SEPT3 plasmid, a gift from Dr. William Trimble (University of Toronto), using the primers 5′- ATTGGATCCA-3′ and 5′- CGCC-TCGAGTCATTCAGCAGTG-3′. The amplified fragment was subcloned into the BamHI and XhoI sites of pET-28a(+). His-SEPT7 was constructed by amplifying the SEPT7 (NP_001107212) sequence using the primers 5′-TCGGGATCCATGTCGGTCAGTGCG-3′ and 5′-GTAAAGCTTTTAAAAGATCTTGCC-3′. The amplified fragment was cloned into the BamHI and HindIII sites of pET-28a(+). To increase the solubility of recombinant SEPT7, His-SEPT7(29-298) was created using the QuikChange II Site-Directed Mutagenesis Kit (Stratagene) to truncate amino acids 1–28 with primers: 5′-CATCATCATCATCACGGCTATGTGGGATTTGC-3′ and 5′-GCAAATCCCACATAGCCGTGATGATGATGATG-3′. Amino acids 229-437 were truncated using the primers 5′-CATGCAGGACTTGAAATAACTCGAGCACCAC-3′ and 5′-GTGGTGCTCGAGTTATTTCAAGTCCTGCATG-3′. The Aurora B expressing plasmid (pRSFduet-GST Δ59N-Aurora B Incenp box), a gift from Dr. Michael Lampson (University of Pennsylvania), was constructed by inserting the GST-tagged sequence of the *Xenopus laevi*s Δ59N-Aurora B into the BamHI and SalI sites of pRSF, and the sequence of the *Xenopus laevi*s Incenp box (aa 790–856) into the BglIII and NotI sites of pRSF.

### Expression and purification of recombinant proteins

Plasmids encoding for recombinant proteins were transformed into *Escherichia coli* BL21(DE3) (Invitrogen). After bacterial cultures reached an OD_600_ of 0.8, protein expression was induced with 0.5 mM IPTG for 16 h at 18°C. Bacteria were centrifuged at 6,000xg for 15 m at 4°C. The cell pellet was resuspended in 10 ml lysis buffer (50 mM Tris-HCl, pH 8.0, 150 mM NaCl, 10% glycerol, 1% Triton X-100, 10 mM imidazole) and lysed by sonication (10 sets of 30 pulses in an ice bath with 30 s interval between each set). The lysate was centrifuged at 20,000xg for 30 m and 4°C. After filtering through a 0.45 µm pore filter, supernatants were loaded onto a column with Ni-NTA or Protino Glutathione Agarose 4B beads, which was equilibrated with 5 ml lysis buffer. The column was washed with wash buffer (50 mM Tris-HCl, pH 8.0, 500 mM NaCl, 10% glycerol, 20 mM imidazole) and the protein was eluted in elution buffer (50 mM Tris/HCl, pH 8.0, 150 mM NaCl, 10% glycerol) containing 250 mM imidazole or 50 mM glutathione. Proteins were analyzed and quantified using 10% SDS-PAGE, and dialyzed overnight.

His-SEPT2 dimers were isolated using an AKTA FPLC system (GE Healthcare) with a Superdex 200 10/300 GL (Amersham Biosciences) gel filtration column. The column was equilibrated with 100 ml of elution buffer (50 mM Tris-HCl pH 8.0, 150 mM NaCl) at a flow rate of 0.5 ml/min. After the equilibration of the column, 500 µl of purified His-SEPT2 (as described before) were loaded onto the column by injecting the protein sample with a syringe in a 500 µl loop. At a flow rate of 0.25 ml/min, His-SEPT2 was eluted in fractions that represented different molecular weights. SDS-PAGE verified the existence of His-SEPT2 in all the fractions where a peak was present and fractions with a molecular weight that corresponded to a SEPT2 dimer were used for assaying protein thermostability.

### Differential scanning fluorimetry (DSF) assay

Differential scanning fluorimetry was performed using a CFX-96 Real Time PCR System (BioRad). SYPRO Orange (Invitrogen) was added at a final concentration of 5× (1∶1,000 dilution) into 50 µl of reaction buffer (50 mM Tris pH 8.0, 50 mM NaCl) containing purified recombinant His-SEPT2 in the presence of DMSO (Sigma-Aldrich), FCF (Sigma-Aldrich), GDP (guanosine 5′-diphosphate; Sigma-Aldrich) or GmpCpp (guanosine-5′-[(a,β)-methylenotriphosphate; Jena Bioscience). Recombinant His-SEPT3, His-SEPT7(29-298) and GST-Aurora B were added in a reaction buffer containing 150 mM NaCl and 50 mM Tris, pH 8.0. SYPRO Orange fluorescence readings were collected with the FRET scan mode at every 0.5°C of temperature increase from 25°C to 75°C; temperature was raised at the rate of 0.5°C/min. Data were collected and processed with the CFC Manager Software using the melt curve function of the software's protocol editing tools. Further analysis and plotting was performed with Microsoft Excel.

### In silico docking

The crystal structures of SEPT2-GDP (PDB ID: 2QNR), SEPT2-GppNHp (PDB ID: 3FTQ), SEPT3-GDP (PDB ID: 3SOP), SEPT7-GDP (PDB ID: 3T5D) and SEPT2/6/7 (PDB ID: 2QAG) were retrieved from RCSB Protein Data Bank (www.rcsb.org/pdb/home/home.do). The residue numbering throughout the manuscript and figures is according to the PDB crystal structure of each protein. FCF structure was obtained from PubChem. Simulations of FCF-septin docking were performed with the AutoDock 4.2.5.1 software running a Lamarckian genetic algorithm (LGA) search method. During docking, the receptor (septin) was set as rigid and the ligand (FCF) was programmed as flexible. The number of docking runs per simulation were 250 and the maximum number of energy evaluations was 2.5 million. The mutation and crossover rates were set to 0.02 and 0.8, respectively, and the population size was 150. Appropriate grid maps were constructed with a grid point spacing of 0.375 Å. Target and ligand preparation, scoring, energy ranking and clustering were performed with AutoDock Tools 1.5.6 (http://autodock.scripps.edu/resources/adt). Visual inspection of poses and septin-FCF interactions were analyzed with the PyMOL Molecular Graphics System, Version 1.5.0.4 Schrödinger, LLC.

### Statistical analysis and sequence alignments

Differential scanning fluorimetry experiments were performed in triplicates and the data are representative of three independent experiments. In silico docking data were statistically analyzed using the AutoDock 4.2.5.1 software. Docking poses were clustered based on their binding free energies and three-dimensional conformations within a root-mean-square-deviation (rmsd) of 2.0 Å. Binding free energies (ΔG) were predicted with the semi-empirical force field function of AutoDock 4.2. FCF-docked conformations were assigned a probability function (P) as previously described [23]. P values served as a measure of binding specificity, indicating the level of confidence in docking pose prediction. Figures show docking poses of the highest P value (lowest binding energy) from their respective clusters of conformations.

Protein sequence alignments were performed with the RCSB PDB Protein Comparison Tool using the Smith-Waterman algorith and text representation for pairwise alignment.

## Results

Currently, high-resolution (∼3 Å) septin structures are available for the GDP-bound SEPT2 (PDB: 2QNR), SEPT3 (PDB: 3SOP) and SEPT7 (PDB: 3T5D), and for SEPT2 bound to the non-hydrolyzable GTP analog GppNHp (PDB: 3FTQ) [11], [13], [14]. Additionally, the crystal structure of the SEPT2/6/7 complex (PDB: 2QAG) has been resolved at a lower resolution of 4 Å [6]. Before docking FCF with these crystal structures, we sought to test whether FCF affects the stability of SEPT2, SEPT3 and SEPT7 similar to previous observations with the SEPT2/6/7 complex [19].

### FCF increases the thermal stability of SEPT2, SEPT3 and SEPT7

To assay for FCF binding and its effects on septin stability, we used a differential scanning fluorescence (DSF) assay that measures protein denaturation as a function of increasing temperature by recording the fluorescence intensity of a dye (SYPRO Orange) which increases when it binds to the hydrophobic regions of a denaturing protein [24], [25]. Thus, DSF reports on protein-ligand interactions that affect protein stability and/or mask protein hydrophobic regions.

**Figure 1:**
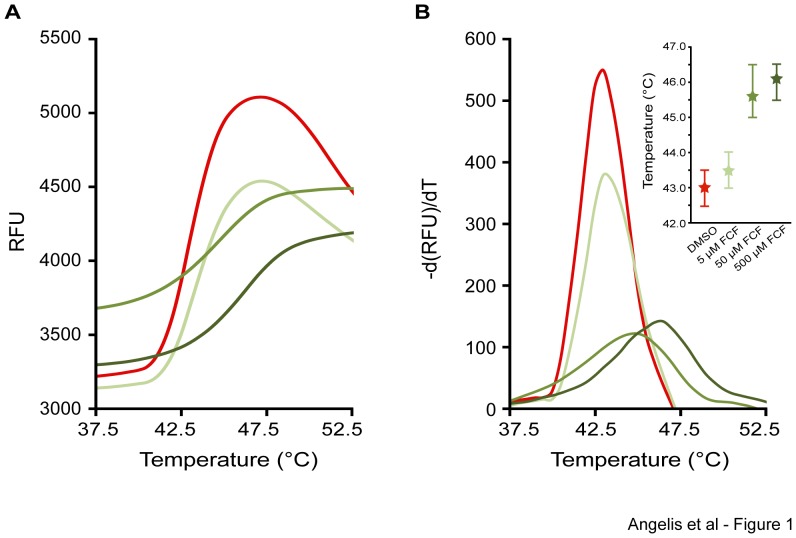
FCF binding induces a shift in the thermal denaturation profile of SEPT2. (A) Curve plots show the fluorescence intensity (relative fluorescence units; RFU) of SYPRO ORANGE as a function of temperature for purified recombinant SEPT2 protein in the presence of DMSO carrier and increasing concentrations of FCF. (B) The negative first derivative of the SYPRO ORANGE fluorescence intensity (RFU) was plotted against temperature. The melting curves illustrate the transition temperatures during the denaturation of SEPT2. Insert shows the median melting temperature of SEPT2 from three independent experiments in the presence of increasing FCF concentrations. Error bars represent the highest and lowest values obtained from three independent experiments.

**Figure 2:**
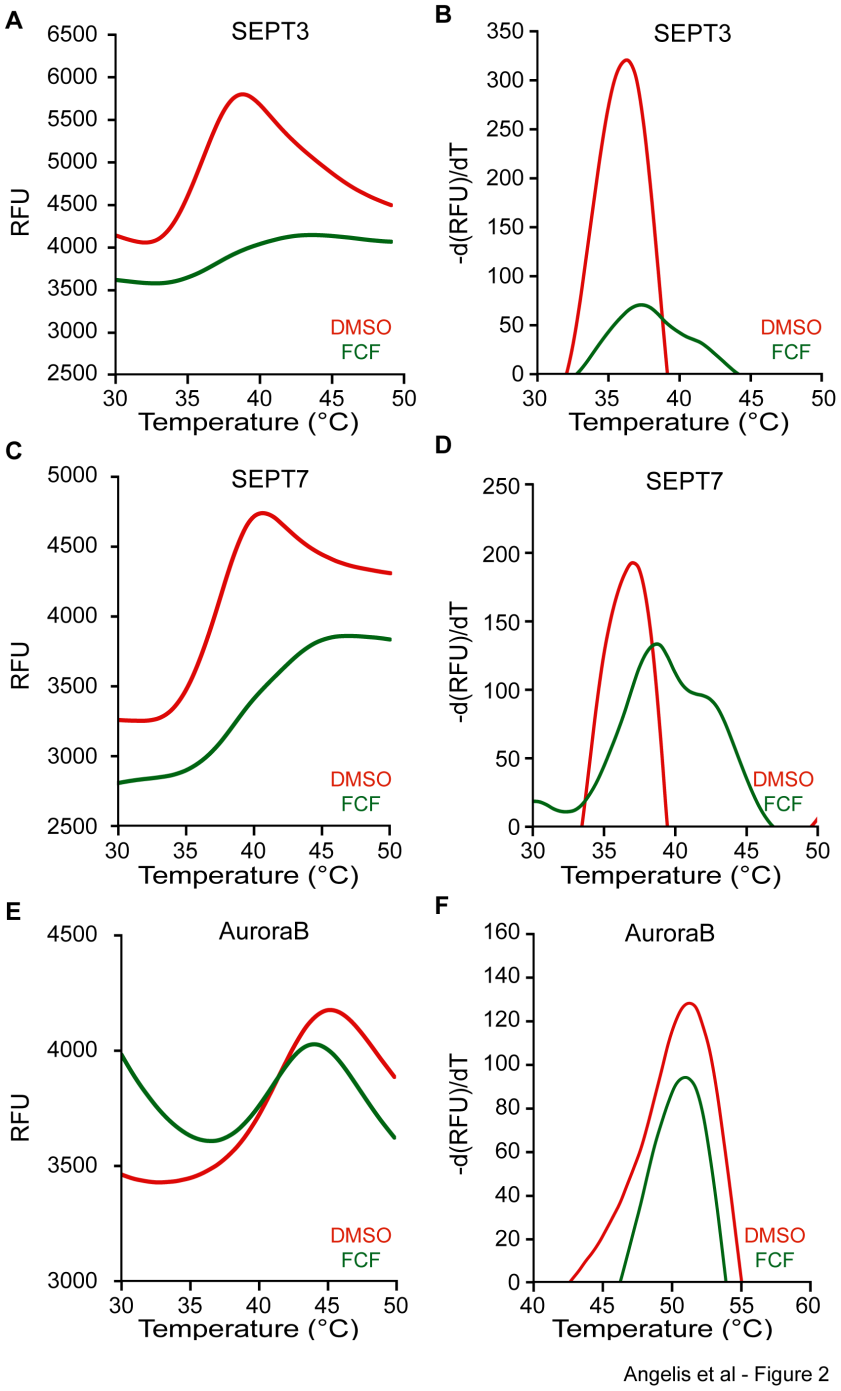
FCF increases the thermal stability of SEPT3 and SEPT7. Differential scanning fluorimetry was performed for purified recombinant His-SEPT3 (A–B), His-SEPT7(29-298) (C–D) and GST-Aurora B (D–E) in the presence of DMSO carrier and FCF (500 µM). Curves show the raw data of fluorescence intensity of SYPRO ORANGE (A, C, E) and the negative first derivative of SYPRO ORANGE fluorescence (B, D, F) plotted against temperature.

We purified recombinant His-tagged SEPT2, SEPT3 and SEPT7(29-298), and performed a DSF assay in the presence of FCF or carrier (DMSO). In the presence of carrier (DMSO), SEPT2 exhibited a denaturation curve with a peak (melting point temperature; Tm) at 43°C (Figure 1). In the presence of increasing FCF concentrations, the denaturation curve of SEPT2 was shifted to higher temperatures (Figure 1B) with melting points increasing to 43.5°C (5 µM FCF), 45.6°C (50 µM FCF) and 46.2°C (500 µM FCF). Similar shifts in the denaturation curve and melting point temperatures were observed for SEPT3 and SEPT7(29-298) (Figure 2A–D), while FCF had no effect on the recombinant Aurora B kinase (Figure 2E–F), a nucleotide-binding kinase used as a negative control. In addition to the increase in melting temperatures, FCF resulted in a decrease in the overall fluorescence of SYPRO Orange, which was particularly noticeable for SEPT2 and SEPT3, and suggested that FCF reduces the accessibility of SYPRO Orange to hydrophobic regions of the denaturing septins. Collectively, these data indicate that FCF binds and increases the thermal stability of SEPT2, SEPT3 and SEPT7.

### *In silico* FCF exhibits differential binding preference for septins

Using the AutoDock program, we simulated FCF binding to the GDP-bound dimeric structures of SEPT2 (2.60 Å resolution), SEPT3 (2.88 Å resolution) and SEPT7 (3.30 Å resolution), the GppNHp-bound SEPT2 dimer (2.90 Å resolution) and the SEPT2/6 G-interface of the SEPT2/6/7 trimer (4 Å resolution). To avoid potential steric hindrances and allow FCF to dock with the entire molecular surface of septins, guanosine nucleotides were removed from their respective structures. We ran 250 independent simulations of FCF binding to each septin structure and the resulting conformations were clustered and sorted based on their predicted binding free energies (**Δ**G).

**Figure 3:**
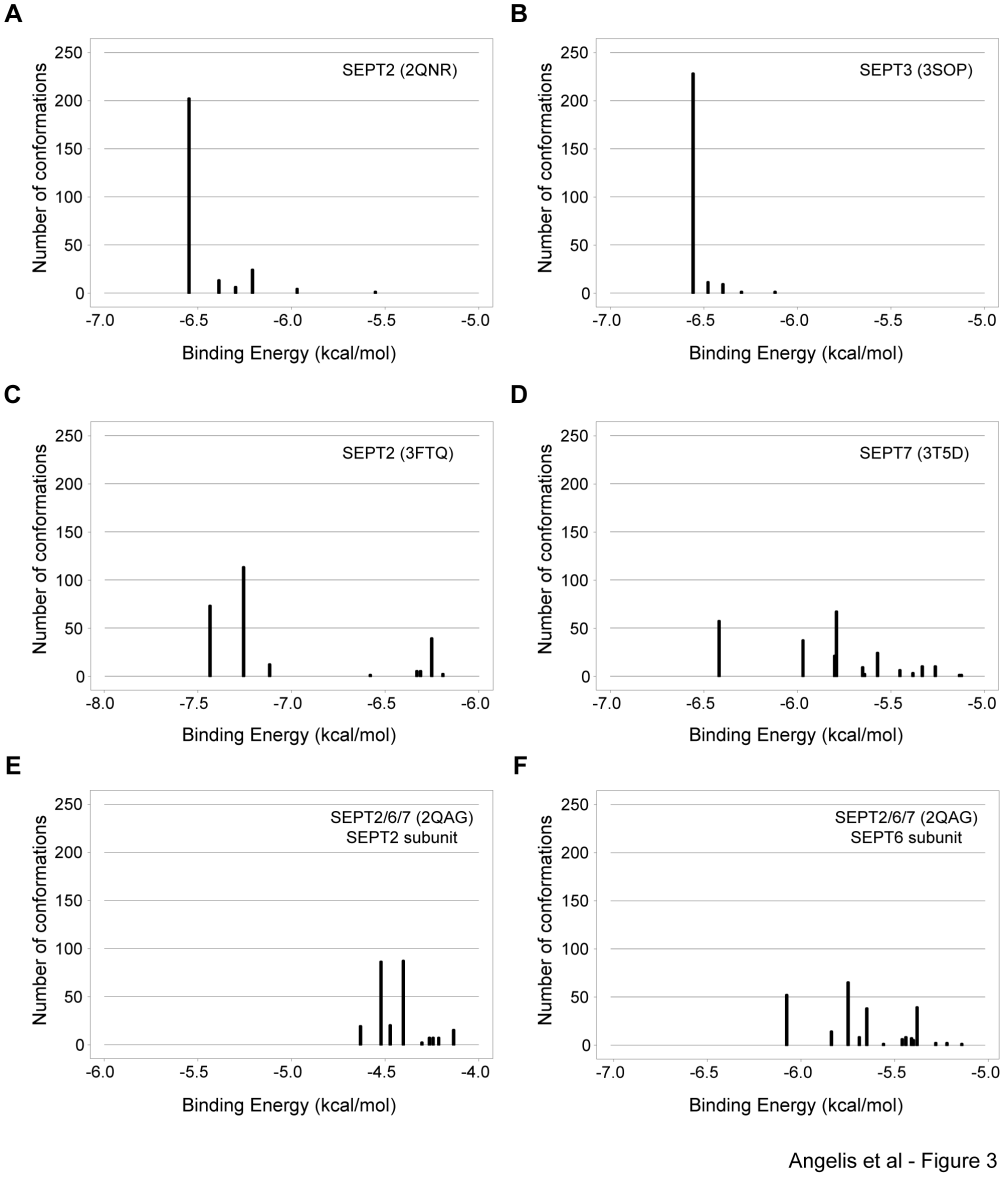
*In silico* FCF exhibits differential binding preference for septins. Histograms show the distribution of binding free energies of 250 conformations of FCF bound to the crystal structures of SEPT2 (A), SEPT3 (B), SEPT2-GppNHp (C), SEPT7 (D), and the SEPT2 (E) and SEPT6 (F) subunits of the SEPT2/6/7 complex. Computational simulations of FCF-septin binding were performed using the AutoDock program after removing the guanosine nucleotides from the septin crystal structures. Each bar represents a cluster of FCF-bound septin conformations of similar docking poses and binding free energies. The number of conformations from each cluster was plotted against the binding free energy of the most energetically preferable conformation (lowest binding free energy).

Docking of FCF with GDP-bound dimers of SEPT2 and SEPT3 showed that 80–90% of the docked poses were clustered around a single conformation (Figure 3A and B). This remarkably high incidence of a single preferred conformation was not observed for any other septin structure. Docking of FCF with the GppNHp-bound SEPT2 dimer yielded two major clusters of docked poses (Figure 3C), which accounted for 30% and 45% of total poses and had the lowest and best binding free energies (∼−7 kcal/mol) compared to all other simulations. FCF docking with the GDP-bound dimer of SEPT7 yielded a variety of different conformations, the preponderance of which were of higher binding free energy (>−6 kcal/mol) than those of SEPT2 and SEPT3 (Figure 3D). Simulations of FCF binding with the SEPT2 and SEPT6 subunits of the SEPT2/6 G interface of the SEPT2/6/7 trimer did not yield any clearly preferred conformations and the docking poses appeared to be energetically unfavorable; note the high binding free energies for the SEPT2 (>−4.5 kcal/mol) and SEPT6 (>−6 kcal/mol) subunits of the SEPT2/6/7 trimer (Figure 3E–F). The latter simulations suffer from the caveat of a lower resolution SEPT2/6/7 structure (4 Å) compared to the homodimeric septin structures, but taken together these results suggest that some septins (e.g., SEPT2, SEPT3) may be a better molecular fit for FCF than others. Thus, FCF appears to possess differential binding preferences for various septins.

### *In silico* FCF is predicted to bind to the nucleotide-binding pockets of SEPT2 and SEPT3

The simulation results for the GDP-bound crystal structures of the SEPT2 and SEPT3 dimers revealed a high preference for a cluster of conformations, in which FCF is bound to the nucleotide-binding pockets of these septins. Notably, 202 out of the 250 docked SEPT2-FCF conformations were within this dominant cluster with an average binding free energy of −6.13±0.42 kcal/mol. Similarly, 228 out of 250 docked SEPT3-FCF conformations belonged to a nucleotide-binding site cluster and had an average binding free energy of −6.47±0.08 kcal/mol.

**Figure 4:**
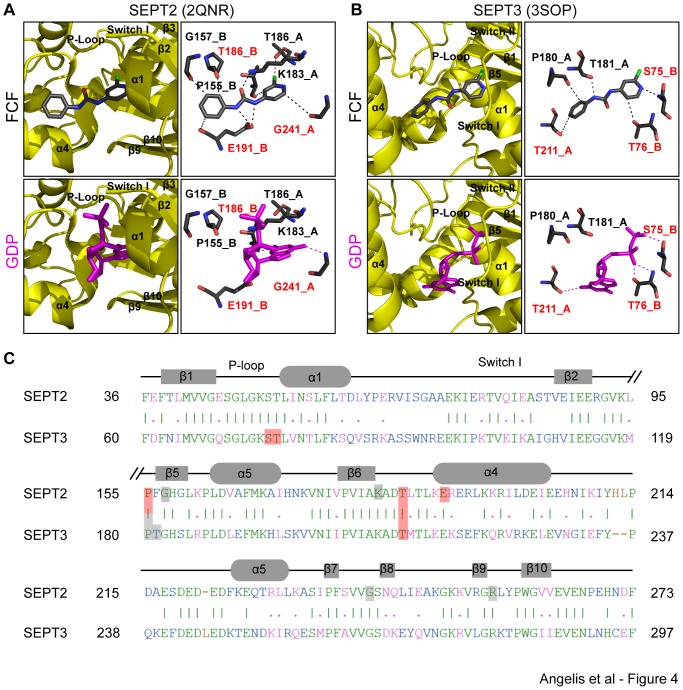
FCF is predicted to interact preferentially with the nucleotide-binding pockets of SEPT2 and SEPT3. (A and B) Ribbon and stick representations show the most energetically favorable conformations of FCF bound to SEPT2 (A) and SEPT3 (B) compared to the crystal structures of the GDP-bound SEPT2 (PDB: 2QNR) and SEPT3 (PDB: 3SOP). Ribbon representations show the position of FCF and GDP with respect to the alpha helices, beta strands, P-loop, and the switch I and switch II regions of the nucleotide-binding pocket of septins. Stick representations depict the amino acids and atomic bonds (dotted lines) underlying the septin interactions with FCF and GDP. Red text denotes amino acids and their corresponding protomers that interact with both FCF and GDP. (C) Alignment of the amino acid sequences of SEPT2 and SEPT3. Common residues that interact with both FCF and GDP are shaded in red and all other amino acids that interact with FCF are shaded in gray. Identical and similar amino acids are shown in green and pink fonts, respectively. Sequence mismatches and insertions/deletions are denoted in blue and brown, respectively.

FCF bound to the nucleotide-binding pocket of SEPT2 by interacting with both of the protomers of the septin dimer (Figure 4A). Sandwiched between protomers A and B within the SEPT2 G interface, FCF is predicted to form hydrogen bonds with Lys-183 and Glu-191 of protomers A and B, respectively. These interactions involve the nitrogen and oxygen atoms of FCF's urea moiety which form strong hydrogen bonds with Glu-191 and Lys-183, respectively. Additional but weaker hydrogen bonds are predicted to occur between the carbon atoms of FCF's phenol ring and the main chain carbonyl groups of Pro-155, Gly-157 and Glu-191 of protomer B (Table 1). In addition, FCF's pyridine ring interacts with the Thr-186 residues of both SEPT2 protomers and Gly-241 of protomer A. Notably, the side chain hydroxyl group of Thr-186 (protomer A) is predicted to form a halogen bond with the chlorine atom of FCF's pyridine ring. The acceptor capability of organic halogen (Cl, F, Br, I) has not been studied in detail in macromolecules [26], but halogen interactions are thought to stabilize protein-ligand interactions [27]. A side-by-side comparison between the FCF- and GDP-bound structures of SEPT2 (Figure 4A) reveals that the FCF-binding residues Thr-186 (protomer B) and Gly-241 (protomer A) interact with the guanine base of GDP, and Glu-191 (protomer B) is bound to the ribose of GDP.

**Table 1:** Summary of septin amino acids that interact with guanine nucleosides and FCF.

SEPT2 - GDP		SEPT2 - GppNHp			SEPT3 - GDP		SEPT7 - GDP		
GDP	FCF	GppNHp	FCF 113*	FCF 73	GDP	FCF	GDP	FCF 57	FCF 37
GLY 47 (A)**	PRO 155 (B)	SER 46 (A)	GLY 49 (A)	SER 46 (A)	LYS 74 (A)	SER 75 (A)	GLY 41 (C)	GLY 43 (C)	GLY 41 (C)
LYS 50 (A)	GLY 157 (B)	LEU 48 (A)	THR 52 (A)	LEU 48 (A)	SER 75 (A)	THR 76 (A)	LEU 42 (C)	THR 46 (C)	SER 45 (C)
SER 51 (A)	LYS 183 (A)	GLY 49 (A)	ASP 185 (A)	SER 51 (A)	THR 76 (A)	PRO 180 (B)	GLY 43 (C)	ASP 178 (C)	THR 46 (C)
THR 52 (A)	THR 186 (B)	LYS 50 (A)	GLU 191 (B)	THR 78 (A)	ASP 210 (A)	THR 181 (B)	LYS 44 (C)	GLU 184 (A)	ASP 94 (C)
ASP 185 (A)	THR 186 (A)	SER 51 (A)	GLY 241 (A)	ASP 101 (A)	THR 211 (B)	THR 211 (B)	SER 45 (C)	GLY 231 (C)	SER 149 (A)
THR 186 (B)	GLU 191 (B)	THR 52 (A)	ARG 256 (A)	THR 102 (A)	GLY 265 (A)		THR 46 (C)	ARG 246 (C)	
GLU 191 (B)	GLY 241 (A)	THR 78 (A)			ARG 280 (A)		SER 149 (A)		
GLY 241 (A)		GLY 104 (A)					ASP 178 (C)		
		LYS183 (A)					THR 179 (A)		
		ASP 185 (A)					GLU 184 (A)		
		THR 186 (B)					GLY 231 (C)		
		GLU 191 (B)					ARG 246 (C)		
		GLY 241 (A)							
		ARG 256 (A)							

*Numbers correspond to the docked poses within the cluster of conformations shown in Figure 3.

**Parentheses indicate the corresponding protomer of each amino acid.

Association of FCF with the nucleotide-binding pocket of SEPT3 involves strong hydrogen bonds between the phenyl-bound nitrogen atom of FCF's urea moiety and the main chain carbonyl group of Thr-181 (protomer A), and between the nitrogen atom of FCF's pyridine ring and the main chain amide of Ser-75 (protomer B; Figure 4B). Weaker hydrogen bonds are predicted to form between two carbon atoms of FCF's phenol ring and the main-chain carbonyl group of Pro-180 and side chain hydroxyl group of Thr-211 of protomer A. In addition, FCF's pyridine ring is predicted to form a hydrogen bond with the side chain hydroxyl group of Thr-76 (protomer B). In the crystal structure of SEPT3, Thr-76 and Ser-75 of protomer B interact with the alpha and beta phosphates of GDP, respectively, and Thr-211 (protomer A) forms a hydrogen bond with the guanine base of GDP.

Taken together, these results suggest that first, FCF may interact preferentially with the nucleotide-binding pockets of SEPT2 and SEPT3, and second, FCF could form hydrogen bonds with residues that come in contact with the guanine base and ribose rings as well as the phosphate groups of GDP. A sequence alignment between SEPT2 and SEPT3 shows that that FCF interacts with residues, which are conserved in both septins (Figure 4C). Pro-155 and Thr-186 of SEPT2 correspond to the Pro-180 and Thr-211 residues of SEPT3. Interestingly, Thr-186/211 interacts with the guanine base of GDP in the crystal structures of both SEPT2 and SEPT3. Thus, FCF and GDP are likely to bind SEPT2 and SEPT3 in a mutually exclusive manner.

**Figure 5:**
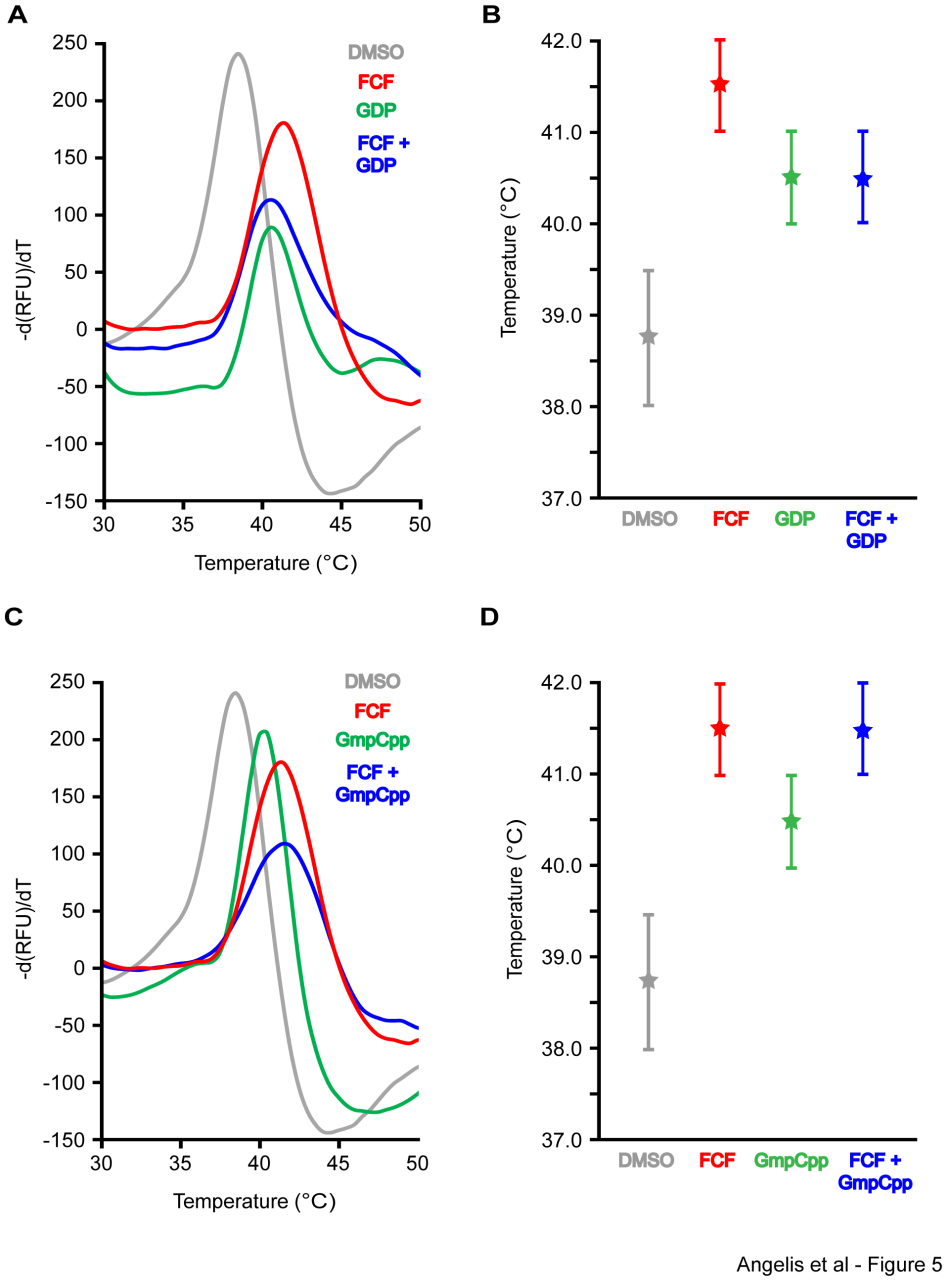
FCF does not affect the thermostability of SEPT2 dimers in the presence of GDP, but increases SEPT2 stability in the presence of GmpCpp. Recombinant His-tagged SEPT2 dimers were purified using size exclusion chromatography and SEPT2 dimer stability was assayed by differential scanning fluorimetry. (A and B) Curves show the negative first derivative of SYPRO ORANGE fluorescence plotted against temperature for SEPT2 dimers in the presence of DMSO (control), FCF (100 µM), GDP (100 µM), and FCF plus GDP (100 µM each). Plot (B) shows the median melting temperature of SEPT2 from three independent experiments. Error bars represent the highest and lowest values obtained from these experiments. (C and D) Curves show the negative first derivative of SYPRO ORANGE fluorescence plotted against temperature for SEPT2 dimers in the presence of DMSO (control), FCF (100 µM), GmpCpp (100 µM), and FCF plus CmpCpp (100 µM each). Plot (D) shows the median melting temperature of SEPT2 from three independent experiments. Error bars represent the highest and lowest values obtained from these independent experiments.

To experimentally test whether FCF and GDP bind SEPT2 in a mutually exclusive manner, we purified recombinant SEPT2 dimers using size exclusion chromatography and assayed for SEPT2 thermostability in the presence of GDP and FCF separately and in combination (Figure 5A–B). In the DSF assay, GDP (100 µM) increased the melting temperature of SEPT2 dimers from 39.0°C to 40.5°C, while FCF (100 µM) caused a larger shift raising the melting temperature to 41.5°C. When combined, FCF and GDP (100 µM each) did not have an additive effect and the melting temperature of SEPT2 dimers was the same with that of SEPT2 in the presence of GDP alone (40.5°C). These, data suggest that GDP occludes the binding of FCF, which cannot exert a stronger stabilizing effect on the structure of SEPT2 than GDP.

### *In silico* binding of FCF to two distinct sites of the nucleotide-binding pocket of the GppNHp-bound structure of SEPT2

Previous work has shown that the GTP- and GDP-bound interfaces of SEPT2 are conformationally different. Presence of GDP in the nucleotide-binding pockets of SEPT2 and SEPT7 triggers conformational changes that stabilize the G interface [6], [11]–[13]. Conversely, presence of GTP is posited to loosen the G interfaces resulting in the dissociation of septin subunits from one another [11], [12]. Because the rate of GTP hydrolysis appears to be inversely proportional to the stability of the G interface [12], nascent septin proteins are likely to associate with GTP prior to their oligomerization, which is favored by their GDP-bound state.

**Figure 6:**
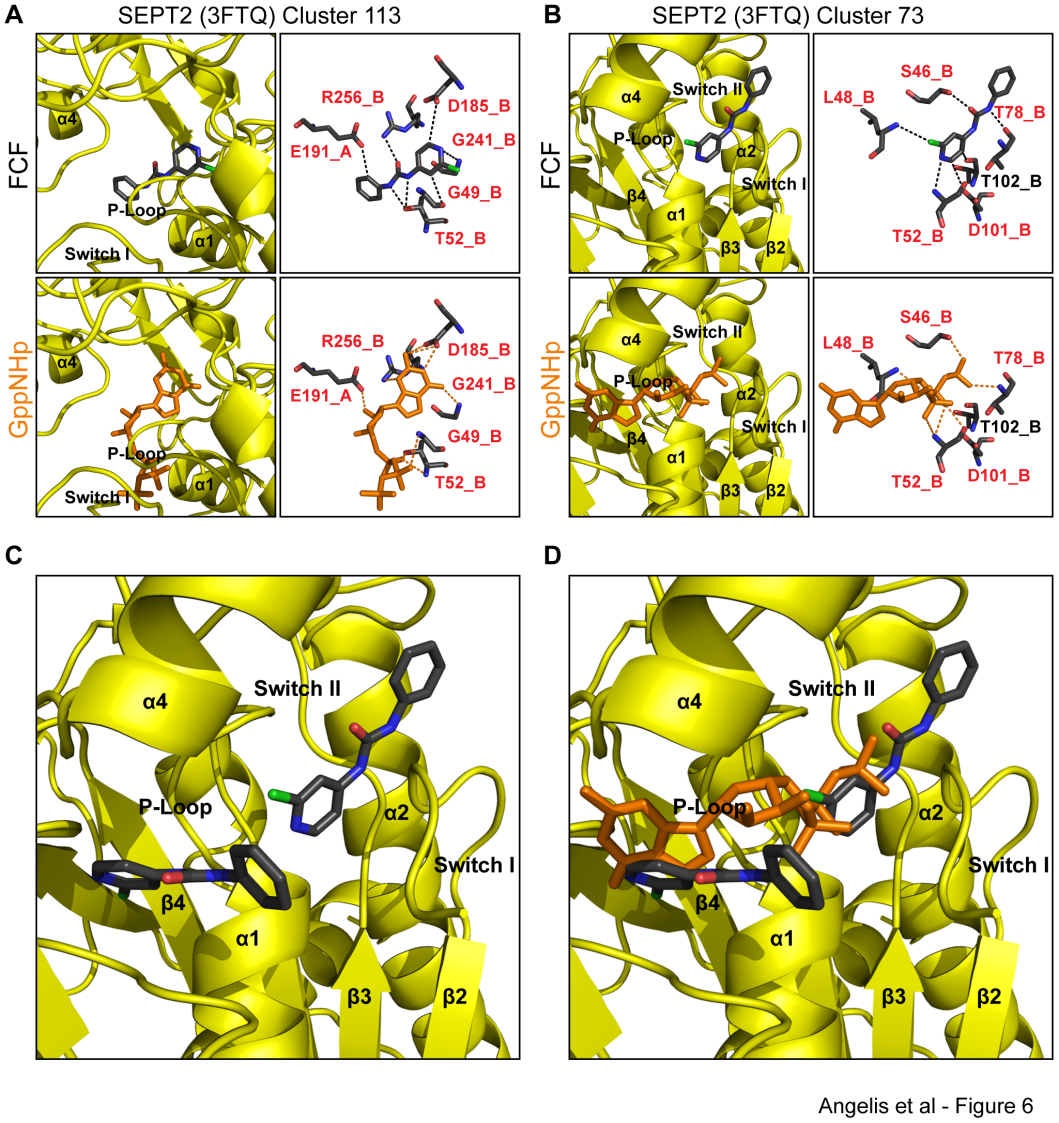
*In silico* binding of FCF to two distinct sites of the nucleotide-binding pocket of SEPT2-GppNHp. (A) Ribbon and stick diagrams show the orientation and atomic interactions of a representative pose of FCF bound to the GppNHp-bound crystal structure of SEPT2 (PDB: 3FTQ) from the cluster of conformations (113 out of 250) with the lowest binding free energy. Red text denotes amino acids and their corresponding protomers that interact with both FCF and GDP. (B) Ribbon and stick representations depict the position and atomic bonds of a representative pose of FCF bound to the GppNHp-bound crystal structure of SEPT2 (PDB: 3FTQ) from the cluster of conformations (73 out of 250) with the second lowest binding free energy. Red letters denote amino acids that interact with both FCF and GDP. (C and D) Ribbon representations show the two conformations of FCF superimposed with the structure of SEPT2 in the absence (C) and presence of GppNHp (D). Note the lack of overlap between the two FCF conformations, which overlap with distinct portions of the GppNHp molecules (guanosine base vs. gamma phosphate).

To gain an insight into how FCF could interact with the more “open” conformation of nascent GTP-bound septins, we simulated docking of FCF to the crystal structure of SEPT2 bound to the GppNHp, a non-hydrolyzable GTP analog. The simulation resulted in two dominant clusters of conformations that occupied two distinct positions on the outer and deep parts of the nucleotide-binding pocket (Figure 6). The average binding free energies of these clusters (−7.19±0.07 kcal/mol and −7.01±0.43 kcal/mol), which were the lowest in all our simulations, indicate that the docked conformations were energetically favorable.

In the first cluster, 113 out of 250 docked conformations are oriented deep in the nucleotide-binding pocket and FCF forms three strong hydrogen bonds with Thr-52, Gly-241 and Arg-256 of protomer B (Figure 6A). Thr-52 and Arg-256 interact with the nitrogen and oxygen atoms of FCF's urea moiety, respectively. Gly-241 interacts with the nitrogen atom of FCF's pyridine ring, whose carbon atoms form weak hydrogen bonds with Gly-49 and Asp-185. An additional hydrogen bond is predicted to occur between the C2 atom of FCF's phenol ring and the side chain carbonyl group of Glu-191 (protomer A). Strikingly, all of FCF's interactions involve amino acids that interact with GppNHp [11]. Of note, Thr-52 is an invariant septin residue that forms a hydrogen bond with the alpha phosphate group of GTP [11], and Asp-185 belongs to the highly conserved GTP specificity motif (AKAD) of septins, which imposes guanine versus adenine nucleotide-binding specificity by forming a canonical double-bifurcated hydrogen bond with the guanine base of GTP (Figure 6A).

In the second cluster of docked poses (73 out of 250), FCF is positioned on the outer side of the GTP-binding pocket (Figure 6B) with an orientation that parallels the position of the phosphate chain of GppNHp (Figure 6D). At this conformation, FCF forms hydrogen bonds with SEPT2 residues that interact exclusively with the phosphate groups of GppNHp. Thr-78, which coordinates with the Mg^2+^ ion and is essential for the catalysis of the gamma phosphate of GTP, forms a strong hydrogen bond with the phenol-bound nitrogen of FCF's urea moiety. Ser-46, which is a P-loop residue that interacts with the gamma phosphate of GTP, is predicted to form a strong hydrogen bond with the oxygen atom of FCF's urea moiety. An additional hydrogen bond occurs between Ser-51, which normally interacts with the beta phosphate of GTP, and the nitrogen atom of FCF's pyridine ring. FCF-SEPT2 binding is further supported by a halogen bond between the main chain amide of Leu-48 and the chlorine atom of FCF, and weaker hydrogen bonds between the carbon atoms of FCF's pyridine ring and Thr-102 and Asp-101 (Table 1).

Superimposing the two conformations of FCF with the structure of SEPT2 shows no overlap (Figure 6C) and demonstrates that in the first cluster of conformations, FCF would interfere with the binding of the guanine, ribose and alpha phosphate groups of GTP, while in the second cluster, FCF would distinctly interfere with the binding of the beta and gamma phosphate groups of GTP, and the hydrolysis of GTP (Figure 6D). In either of these two conformations, FCF is predicted to compete for binding with GTP. To test this experimentally, we assessed the effects of GmpCpp, a non-hydrolyzable GTP analog, and FCF on the thermostability of SEPT2 dimers (Figure 5C–D). In the presence of GmpCpp (100 µM), the melting temperature of SEPT2 increased from 39°C to 40.5°C. In contrast, FCF increased the melting peak of SEPT2 dimers to 41.5°C. In the presence of both GmpCpp and FCF, the melting temperature SEPT2 dimers did not surpass 41.5°C, which was the temperature recorded in the presence of FCF (Figure 5D). Lack of a synergistic effect suggests that GmpCpp and FCF do not bind SEPT2 simultaneously and may indeed compete with one another. Given that GTP loosens the G interface of septin molecules and induces their dissociation [11], [12], FCF may bind SEPT2 molecules more readily imposing its stabilizing effects.

### *In silico* docking of FCF with the crystal structure of SEPT7 suggests that the sites of FCF-binding are conserved between SEPT7 and SEPT2

In contrast to the highly favored conformations obtained in the simulations of FCF binding with SEPT2 and SEPT3, docking of FCF with SEPT7 yielded a number of different conformations with higher binding free energies. We focused our analysis on the two most energetically favorable clusters of conformations, whose lowest free binding energies were −6.42 kcal/mol and −5.95 kcal/mol (Figure 3D) and accounted for 23% and 15% of the docked poses, respectively. Surprisingly, these conformations were similar to those of FCF on the outer and deep parts of the nucleotide-binding pocket of GppNHp-bound SEPT2.

**Figure 7:**
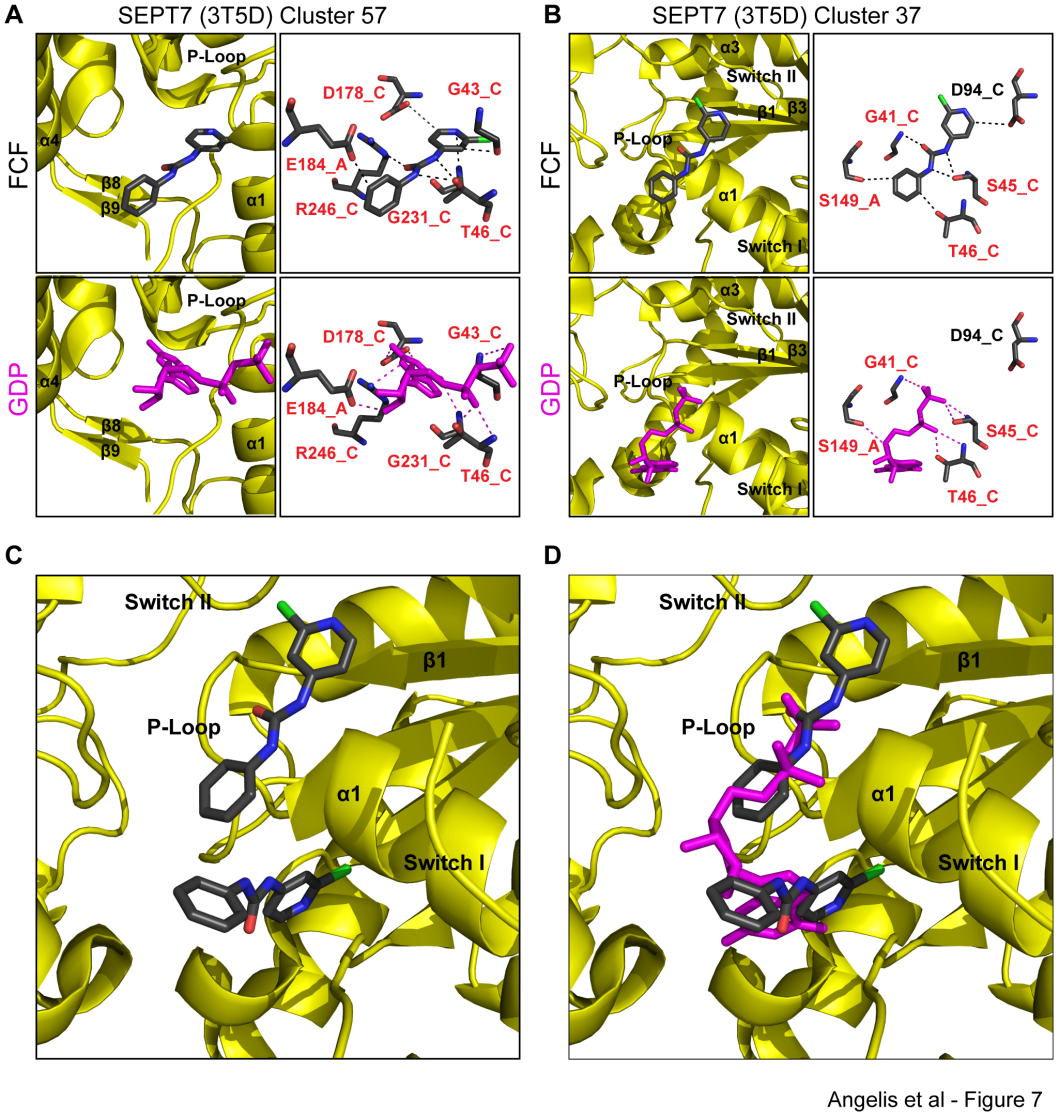
FCF is predicted to bind to the outer side or deep into the nucleotide-binding pocket of SEPT7 in a similar fashion to its interaction with SEPT2-GppNHp. (A) Ribbon and stick diagrams show the orientation and atomic interactions of a representative pose of FCF bound to SEPT7 (PDB: 3T5D) from the cluster of conformations (57 out of 250) with the lowest binding free energy. Red text denotes amino acids and their corresponding protomers that interact with both FCF and GDP. (B) Ribbon and stick representations depict the position and atomic bonds of a representative pose of FCF bound to SEPT7 (PDB: 3T5D) from the cluster of conformations (37 out of 250) with the second lowest binding free energy. Red text denotes amino acids and their corresponding protomers that interact with both FCF and GDP. (C–D) Ribbon representations show the two lowest energy conformation of FCF superimposed with the nucleotide-binding pocket of SEPT7 in the absence (C) and presence of GDP (D). Similar to the dominant conformations of FCF with the nucleotide-binding pocket of SEPT2-GppNHp (Figure 6C–D), FCF molecules occupy two spatially distinct regions of SEPT7 and overlap with distinct moieties of GDP (guanosine base vs. phosphate chain).

**Figure 8:**
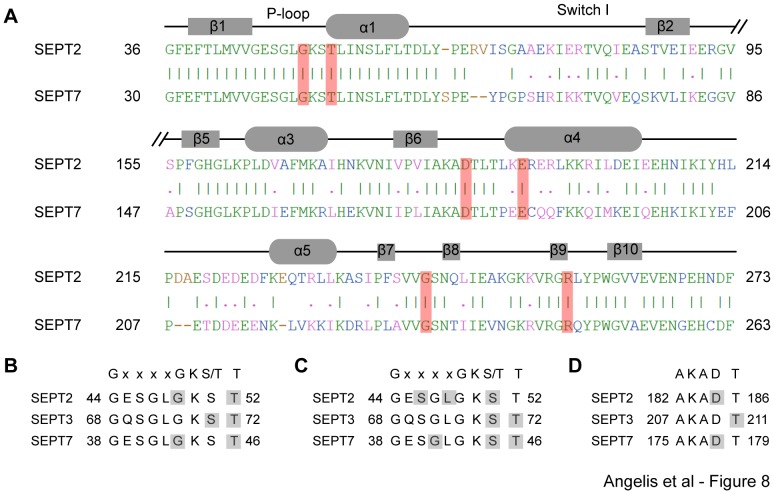
*In silico* FCF interacts with highly conserved septin residues and signature motifs. (A) Sequence alignment highlights the conserved amino acids (shaded in red) that mediate the interaction of FCF with the deep end of the nucleotide binding pockets of SEPT2 (PDB: 3FTQ) and SEPT7 (PDB: 3T5D). Identical and similar amino acids are shown in green and pink fonts, respectively. Sequence mismatches and insertions/deletions are denoted in blue and brown, respectively. (B and C) Sequence alignments of the Walker A motif GxxxxGKS/T and the immediately following threonine, an invariant septin residue, highlight the amino acids (shaded in gray) that interact with FCF at the deep end (B) and outer side (C) of the nucleotide-binding pockets of SEPT2 (PDB: 3FTQ), SEPT3 (PDB: 3SOP) and SEPT7 (PDB: 3T5D). (D) Sequence alignments of the GTP-binding specificity motif AKAD and the following threonine residue highlight the amino acids (shaded in gray) that interact with FCF at the deep end of the nucleotide-binding pockets of SEPT2 (PDB: 3FTQ), SEPT3 (PDB: 3SOP) and SEPT7 (PDB: 3T5D).

In the cluster of conformations with the lowest average binding energy (−5.87±0.55 kcal/mol), FCF is positioned deep in the nucleotide-binding pocket of SEPT7 with its phenol ring and urea moiety occupying the site of the guanine base and ribose ring of GTP. Similar to the conformation of the SEPT2-bound FCF, the nitrogen and oxygen atoms of FCF's urea moiety form hydrogen bonds with Thr-46 and Arg-246 of SEPT7 (Figure 7A), which correspond to Thr-52 and Arg-256 of SEPT2 (Figure 6A). In an identical fashion with Gly-241 of SEPT2, Gly-231 forms a strong hydrogen bond with the nitrogen atom of FCF's pyridine ring. Asp-178 of the conserved GTP-binding specificity motif (AKAD) of septins is predicted to form a hydrogen bond with FCF's pyridine ring, which also interacts with the conserved P-loop residue Gly-43 (Gly-49 in SEPT2). Similar to Glu-191 of SEPT2, Glu-184 is hydrogen-bound to a carbon atom from FCF's phenol ring. Strikingly, FCF interacts with six residues of SEPT7, which are completely conserved between SEPT7 and SEPT2 (Figure 8A), and are the same amino acids that mediate FCF binding to SEPT2.

In the cluster of conformations with the second lowest binding free energy (−5.72±0.25 kcal/mol), FCF is positioned on the outer side of the nucleotide-binding pocket with its phenol ring and half of its urea moiety occupying the space where the alpha and beta phosphate groups of GDP normally reside (Figure 7B and D). Ser-45 of the septin P-loop Walker A motif GxxxxGKS/T (Ser-51 in SEPT2) forms a double bifurcated hydrogen bond with the N atoms of FCF's urea moiety. Gly-41, another conserved residue of the P-loop Walker A motif (Gly-47 in SEPT2), interacts with the oxygen atom of FCF. Weaker hydrogen bonds are predicted between the carbon atoms of FCF's pyridine and phenol rings and Asp-94, Ser-149 and Thr-46. The latter amino acid is an invariant septin residue that comes in contact with the alpha phosphate of GDP. These interactions indicate that FCF binds to the outer side of the nucleotide-binding pocket by forming strong hydrogen bonds with residues of a highly conserved P-loop domain that interacts with the phosphate groups of the guanosine nucleotide.

Similar to FCF binding to SEPT2 (Figure 6C), the two most energetically favored conformations of the SEPT7-bound FCF occupy two distinct sites of the nucleotide pocket without overlapping spatially (Figure 7C). The orientations of these conformations relative to the position of the guanosine nucleotide are also very similar between SEPT7 and SEPT2 (Figures 6D and 7D), and suggest that FCF may interfere with the binding of the adenosine and/or phosphate groups of GTP.

## Discussion

FCF is a synthetic urea derivative with strong cytokinin activity in plants [17]. Deemed as an agent with relatively short half-life and low *in vivo* toxicity, FCF is a government-approved fertilizer used in fruit horticulture. After the fortuitous discovery of its effects on the septin cytoskeleton [18], FCF has become a pharmacological tool for the study of septin functions. FCF affects septin organization and dynamics in organisms as diverse as yeast, frogs and mammals. FCF treatments have been reported to phenocopy the effects of septin-depletion affecting biological processes such as cell division and cell motility [19], glucose uptake [28], store operated calcium influx [29], ciliogenesis [30] and embryonic gastrulation [31]. Recent work also showed that FCF enhances septin-mediated degradation of the hypoxia inducible factor-1a and decreases the proliferation of prostate cancer cells [32]. While in vitro biochemical studies indicate that FCF affects directly the organization of purified recombinant septins [19], FCF has been shown to bind a cytokinin-binding protein from mung bean seedlings [33]. Septins are absent from plants, but the possibility of FCF targeting proteins other than septins in non-plant species is a valid concern that cannot be ruled out. Thus, understanding how FCF binds and affects septins is important not only for its present use as a small molecule inhibitor, but also for the future development of septin-targeting compounds.

Employing *in silico* computational approaches, which are commonly used in drug screening and structure-based drug design, we sought to gain an insight into the structural mechanism of FCF recognition by septins. Docking of FCF with all the crystal structures of septins predicts that FCF has a preference for the GTP-binding pocket. We found that the binding free energies and affinity of FCF can vary *in silico* depending on septin paralog, suggesting that FCF may access or fit the nucleotide pockets of some septins better than others. Beyond this differential preference, however, our results suggest a conservation in the structural features of binding. Strikingly, FCF is predicted to interact with signature septin motifs such as the G1 Walker A motif (GxxxxGKS/T) and the G4 GTP-binding specificity motif AKAD (Figure 8B–D), and a highly conserved threonine residue that catalyzes the hydrolysis of GTP. These interactions suggest that FCF mimics nucleotide binding, interfering with GTP binding and possibly hydrolysis.

Collectively, our simulations predict that FCF could bind deep into or on the outer side of the nucleotide-binding pocket. At either position, FCF interacts with the P-loop between strand β1 and helix a1, and an invariant threonine residue that immediately follows the GxxxxGKS/T motif of the P-loop. This appears to be the most salient feature of FCF binding as it is conserved *in silico* in the interactions of FCF with three different septin paralogs (SEPT2, SEPT3 and SEPT7). When FCF is bound to the outer side of the nucleotide-binding pocket, the P-loop is predicted to provide half or more of the amino acids that FCF binds. Thus, presence of FCF would interfere with binding of the phosphate groups of GDP/GTP to the P-loop of septins. It is unclear if FCF can interact with the outer side of the nucleotide-binding pocket in the presence of GTP, but if this were true, FCF would interfere with both the binding and hydrolysis of the gamma phosphate of GTP. In support of this possibility, docking of FCF with the GppNHp-bound structure of SEPT2 showed that FCF forms a strong hydrogen bond with Thr-78, which is an essential residue for the mechanism of GTP hydrolysis by septins. When FCF is positioned deeply into the nucleotide-binding pocket, the aspartate residue from the G4 AKAD motif and the immediately following threonine provide a conserved point of contact with FCF (Figure 8D). In the case of SEPT2 and SEPT7, glutamate, glycine and arginine residues are predicted to provide additional points of interaction with FCF downstream of the AKAD motif (Figure 8A; Table 1). These amino acids interact with the adenine base or the ribose ring of guanosine, suggesting that FCF would block the binding of the guanosine moiety of GTP. Overall, FCF may target septins by interacting with highly conserved domains that underlie the mechanism of GTP-binding and hydrolysis.

Previous studies have shown that FCF treatments enhance the assembly of higher order septin structures *in vitro* and dampen the exchange of subunits on filamentous polymers in live cells [19], [20]. If FCF and GTP compete for binding to septins, then how does FCF stabilize and amplify the formation of septin filaments? Because the GDP-bound interface of dimeric and oligomeric septins is rather stable and GDP turnover is very slow [12], [15], [34], [35], it is difficult to envisage how FCF would affect the dynamics of pre-existing polymers. One possibility is that FCF binds to the outer side of the nucleotide pocket of polymeric septins and stabilizes them in the presence of GDP. This, however, might not be possible because the phosphate groups of GDP will be masking all the P-loop residues needed for FCF binding. Indeed, our data indicate that FCF cannot increase the thermostability of SEPT2 dimers in the presence of GDP (Figure 5A–B). An alternative possibility is that FCF targets nucleotide-free or GTP-bound subunits with a less stable and more open G-interface and thus, of a higher GTP turnover rate. Consistent with this possibility, FCF was able to increase the thermostability SEPT2 in the presence of the GTP analog GmpCpp (Figure 5C–D). On septin filaments, therefore, FCF could target the constitutively GTP-bound members of the SEPT6 group and septins positioned at the ends of septin polymers. FCF would bind these septin subunits by mimicking GTP, triggering conformational changes that further stabilize the G and NC interfaces, and decrease dissociation rates.

In summary, our results provide an insight into the structural features of FCF binding to septins and offer a blueprint for the structure-based design and screening of septin-targeting compounds. *In silico* FCF is predicted to interact with signature septin domains, motifs and residues, which are highly conserved in septin homologs and paralogs across species. While our findings are largely based on theoretical models and have yet to be proven experimentally, they provide an explanation for the broad specificity of FCF, which has been shown to affect septin organization and functions in evolutionarily diverse organisms. Future X-ray crystallographic studies will elucidate the precise mechanism of FCF recognition by septins.
